# A new fusion neural network model and credit card fraud identification

**DOI:** 10.1371/journal.pone.0311987

**Published:** 2024-10-28

**Authors:** Shan Jiang, Xiaofeng Liao, Yuming Feng, Zilin Gao, Babatunde Oluwaseun Onasanya

**Affiliations:** 1 School of Computer Science and Engineering, Chongqing Three Gorges University, Wanzhou, Chongqing, China; 2 College of Computer Science, Chongqing University, Chongqing, China; 3 Key Laboratory of Intelligent Information Processing and Control, Chongqing Three Gorges University, Wanzhou, Chongqing, China; 4 Department of Mathematics, University of Ibadan, Ibadan, Nigeria; University of Mauritius, MAURITIUS

## Abstract

Credit card fraud identification is an important issue in risk prevention and control for banks and financial institutions. In order to establish an efficient credit card fraud identification model, this article studied the relevant factors that affect fraud identification. A credit card fraud identification model based on neural networks was constructed, and in-depth discussions and research were conducted. First, the layers of neural networks were deepened to improve the prediction accuracy of the model; second, this paper increase the hidden layer width of the neural network to improve the prediction accuracy of the model. This article proposes a new fusion neural network model by combining deep neural networks and wide neural networks, and applies the model to credit card fraud identification. The characteristic of this model is that the accuracy of prediction and F1 score are relatively high. Finally, use the random gradient descent method to train the model. On the test set, the proposed method has an accuracy of 96.44% and an F1 value of 96.17%, demonstrating good fraud recognition performance. After comparison, the method proposed in this paper is superior to machine learning models, ensemble learning models, and deep learning models.

## Introduction

In recent years, China’s credit cards have made significant progress. Credit cards play a huge role in the market economy and national economy. With the rapid development of credit cards, the fraudulent behavior of credit cards is also increasing. Credit card fraud [1] refers to fraudulent behavior committed with the purpose of illegally occupying resources, in the process of purchasing, using, and consuming credit cards, in violation of relevant bank regulations or national laws related to credit card issuance.

Common credit card fraud behaviors [2] include: (1) Using forged or invalidated credit cards for consumption, overdraft, or fraud. (2) Pretending to be someone else’s credit card for consumption, overdraft, or fraud. (3) Intentionally or maliciously overdrawing a credit card and long-term denial. The fraudulent behavior of credit cards poses a huge threat to the safe and stable development of banks and the financial industry. Therefore, researching and constructing a machine learning model and method that can accurately identify credit card fraudulent transactions is of great significance for the scientific management and rapid and healthy development of credit card business.

In order to avoid the huge risks brought by credit card fraud [3], the banking and financial industries urgently need a credit card fraud identification system that can effectively identify whether there is fraudulent behavior in transaction records.

The current credit card fraud identification method still has the problem that accuracy and F1 score is not high enough. At the same time, there is a great demand for a method with low time complexity and low space complexity. At present, neural networks are a very popular direction, and deep feedforward networks and wide neural networks can optimize credit card fraud detection models to a certain extent, improving their detection accuracy and correctness. The application of these two networks in credit card fraud detection is still relatively small. The method proposed in this paper not only provides a new model for this problem, but also improves the recognition accuracy.

This article will propose a fusion neural network that combines deep neural networks and width neural networks, and apply it to credit card fraud identification. Due to the excellent performance of neural networks [4] in binary classification problems, this paper will construct a credit card fraud recognition model based on neural networks. The first method is to deepen the layers of the neural network to improve the prediction accuracy of the model. The second method is to increase the hidden layer width of the neural network to improve the prediction accuracy of the model. Finally, by combining deep neural networks and wide neural networks, a new fusion neural network model is proposed and applied to credit card fraud identification. The model in this article can effectively improve the accuracy of credit card fraud identification and provide a model with good time and space complexity

In the following chapters, this article first introduces the current research status of credit card fraud identification. In the method section of the article, this paper will introduce the Independent variable and dependent variable respectively, deep neural network, width neural network, fusion neural network, model training, model evaluation criteria. In the numerical experiment section, this paper will discuss data set, analysis of influencing factors, experimental results, comparison with other models, time and space complexity analysis. The last part is a conclusion.

## Related works

At present, credit card recognition technology mainly relies on machine learning [5,6] methods. Machine learning algorithms can be divided into two categories: Supervised learning [7] and Semi-Supervised Learning. The methods of machine learning mainly include: Logistic regression [8], K Nearest Neighbor (KNN) [9], Decision tree [10], Bayesian network [11], support vector machine (SVM) [12].

At present, many scholars have conducted in-depth research on credit card fraud detection based on machine learning methods, including: algorithm based on decision tree and boolean logic [13], cost sensitive decision tree algorithm [14], risk induced cost sensitive minimal Bayesian algorithm [15], parallel fuzzy neural network [16], a framework for detecting potential fraudulent transactions in credit card transactions mining based on CNN [2], support vector machine model with Spark [17]. Some scholars have introduced semi supervised learning [18] and ensemble learning [19] into the problem of credit card fraud identification.

Some scholars have improved the credit card scoring system itself to obtain a better credit card scoring model to increase the success rate of fraud detection. They used a combination of logistic regression and weighted evidence to construct a hybrid credit scoring model, which improved the accuracy and predictive power of credit scoring and reduced the risk of credit fraud [20]. In the realm of deep learning, scholars have used supervised learning algorithms such as artificial neural networks (ANN), support vector machines (SVM), and deep neural networks (DNN) to predict fraud risks, achieving high recognition accuracy [21,22]. In order to discover the patterns of credit card fraud transactions, fuzzy neural networks are used, and it is pointed out that fuzzy neural networks can discover fraud patterns in a short period of time. Some scholars use self-encoder neural networks to detect credit card fraud [23].

At present, some scholars have conducted in-depth research on credit card fraud detection using ensemble learning methods [24,25]. Through supervised learning on the dataset using random forest, logistic regression, and AdaBoost classifiers, the detection accuracy was greatly improved, and a research idea was proposed to combine LSTM prediction with random forest based on HMM [26,27]. Some scholars have designed a credit card fraud prediction model based on clustering analysis and ensemble support vector machines, using the idea of ensemble learning to further address the imbalance of data and improve the recognition of minority classes by classifiers. Finally, testing is conducted [28].

In the field of heuristic learning, many authors have done relevant work. For example, some scholars have proposed tuning machine learning models using a group search firefly algorithm for fraud detection [29]. Some scholars have proposed metaheuristics based hyperparameters optimization for credit card fraud detection [30]. Some scholars have used firefly metaheuristics to optimize the adaboost algorithm [31]. Some scholars have optimized the feature selection for credit card fraud identification based on the Oppositional Cat Swarm Optimization method [32]. Some scholars optimize the XGBoost model based on heuristic optimization methods to identify fraud [33]. Some scholars have constructed the adaboost algorithm based on the SNS heuristic optimization method [34]. In general, the current predictive model of heuristic optimization is a popular trend. These methods can optimize fraud detection to a certain extent.

In the research on credit card fraud identification based on neural networks, the current methods mainly include: Neural Network Ensemble with Feature Engineering [35], Stacked Sparse Autoencoder Approach [36], Method based on deep convolutional neural network [37], Graph Neural Network-based Method [38], Method based on competitive graph neural network [39]. These methods can promote the application of neural networks in credit card fraud identification.

## Fraud identification methods

**Fig 1:**
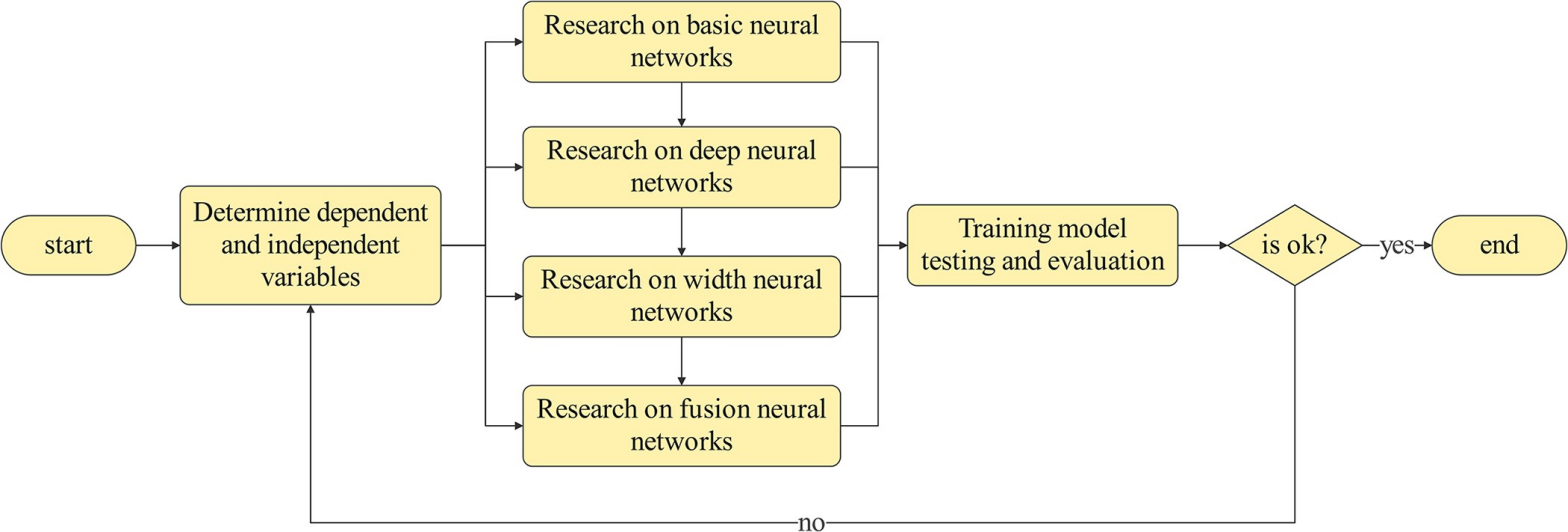
Flow chart for constructing a credit card fraud model based on neural networks.

In this article, a credit card fraud recognition model based on deep neural networks and wide neural networks is proposed. In order to establish this model, this paper must determine the dependent and independent variables of the credit card fraud identification problem, build a deep neural network model, build a width neural network model, study the basic principle and structure of the fusion neural network model that combines the deep neural network and the width neural network, then train the these model, and finally use the test set to test and evaluate. The flowchart for modeling is shown in Fig 1.

### Independent variable and dependent variable

The content of this article is credit card fraud identification. Credit card fraud identification is to predict whether a credit card is fraudulent based on several attributes and transaction amounts of each transaction. In this article, the *j*-th attribute of the *i*-th transaction is defined as:

$$v_{i,j},i=1,2,\cdots ,m,j=1,2,\cdots ,n$$ (1)

This article uses all 28 variables and transaction amount in the data set as input variables to predict whether there is fraudulent behavior. The model we want to establish is as follows:

$$y_{i}=f(v_{i,1},v_{i,2},\cdots ,v_{i,n},a_{i})$$ (2)

where value of *y*_*i*_ is 0 or 1, if *y*_*i*_ = 1, it indicates that the transaction is fraudulent, and if *y*_*i*_ = 0, it indicates that the transaction is not fraudulent. *f* is the model we want to establish, where *v*_*i*,*j*_ represent the values of the *i*-th transaction and the *j*-th attribute. *a*_*i*_ is the amount of the *i*-th transaction. In order to construct the training data set for the model, this paper record the output variables of the model training set as:

$$Y={\left[y_{1},y_{2},\cdots ,y_{m}\right]}^{T}$$ (3)

where *Y* is an m-dimensional column vector, this paper note the input variable of the model training set as:

$$V=[\begin{array}{cccc} v_{1,1} & \ldots & v_{1,n} & a_{1}\\ v_{2,1} & \ldots & v_{2,n} & a_{2}\\ \ldots & \ldots & \ldots & \ldots \\ v_{m,1} & \ldots & v_{m,n} & a_{m} \end{array}]$$ (4)

This paper use *Y* and *V* to obtain this model parameters, and then use this model to predict whether there is fraudulent behavior in credit card transactions, and test the accuracy of the model on the test set.

### Deep neural network

The credit card fraud recognition in this article is a typical nonlinear binary classification problem, which is applicable to the nonlinear classification problem in the input space. The input variables for this problem are the 28 attributes and transaction amount of the credit card, with outputs of 0 or 1, where 0 represents normal transactions and 1 represents fraudulent transactions. Neural network models can effectively handle nonlinear classification problems because they can characterize any nonlinear function. In order to achieve better fitting results, this article deepens the layers of the neural network to improve the model’s generalization ability. The neural network in this article consists of 6 hidden layers and 6 relu layers [20]. Finally, it is output as a category variable through the softmax layer. The forward calculation formula for deep neural networks is as follows:

$$x_{k}^{\left(1\right)}=f_{k}^{\left(1\right)}(v_{i},a_{i})=relu(\sum_{j=1}^{n_{1}}\omega_{k,j}^{\left(1\right)}v_{i,j}+\omega_{k,n_{1}+1}^{\left(1\right)}a_{i}+\omega_{k,n_{1}+2}^{\left(1\right)})$$

$$x_{k}^{\left(2\right)}=f_{k}^{\left(2\right)}(x_{i}^{\left(1\right)})=relu(\sum_{j=1}^{n_{2}}\omega_{k,j}^{\left(2\right)}x_{i,j}^{\left(1\right)}+\omega_{k,n_{2}+1}^{\left(2\right)})$$

**Fig 2:**
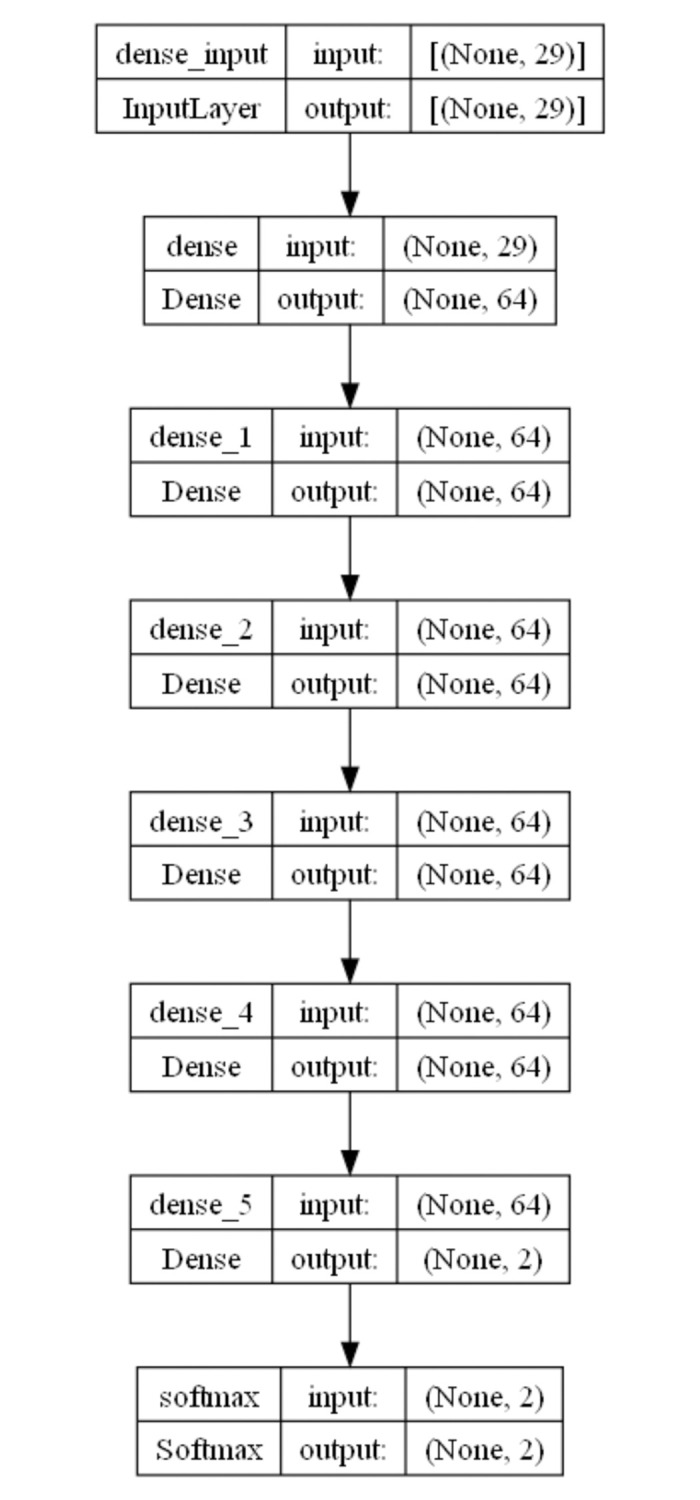
Structure diagram of deep neural network.

……

$$x_{k}^{\left(6\right)}=f_{k}^{\left(6\right)}(x_{i}^{\left(5\right)})=relu(\sum_{j=1}^{n_{6}}\omega_{k,j}^{\left(6\right)}x_{i,j}^{\left(5\right)}+\omega_{k,n_{6}+1}^{\left(6\right)})$$

$$P(y_{i}=l)=\frac{\exp(x_{l}^{\left(6\right)})}{\sum_{k=1}^{L}\exp(x_{k}^{\left(6\right)})}$$

$$l=\arg\;\underset{j}{\max}\;p(y_{i}=j)$$ (5)

where *ω*^(1)^ is the coefficient of the first layer model, *ω*^(2)^ is the coefficient of the second layer model, *ω*^(6)^ is the 6-th layer model coefficient. *n*_1_ is the number of neurons in the first layer, *n*_2_ is the number of neurons in the second layer, *n*_6_ is the number of neurons in the 6-th layer, *v* is the input variable, *a* is the amount of each transaction, *v*_*i*_ is the data in the training set. *x*^(1)^ is the output of the first hidden layer, *x*^(2)^ is the output of the second hidden layer, *x*^(6)^ is the output of the 6-th hidden layer, *f* is the nonlinear transformation function of the hidden layer, *L* is the number of categories (Take *L* as 2 in this article), *b* is the bias term. Fig 2 is the structural diagram of the deep neural network. The *relu* is a nonlinear activation function. Its definition is as follows:

$$relu(x)=\{\begin{array}{l} x,x\geq 0\\ 0,x<0 \end{array}$$ (6)

### Width neural network

**Fig 3:**
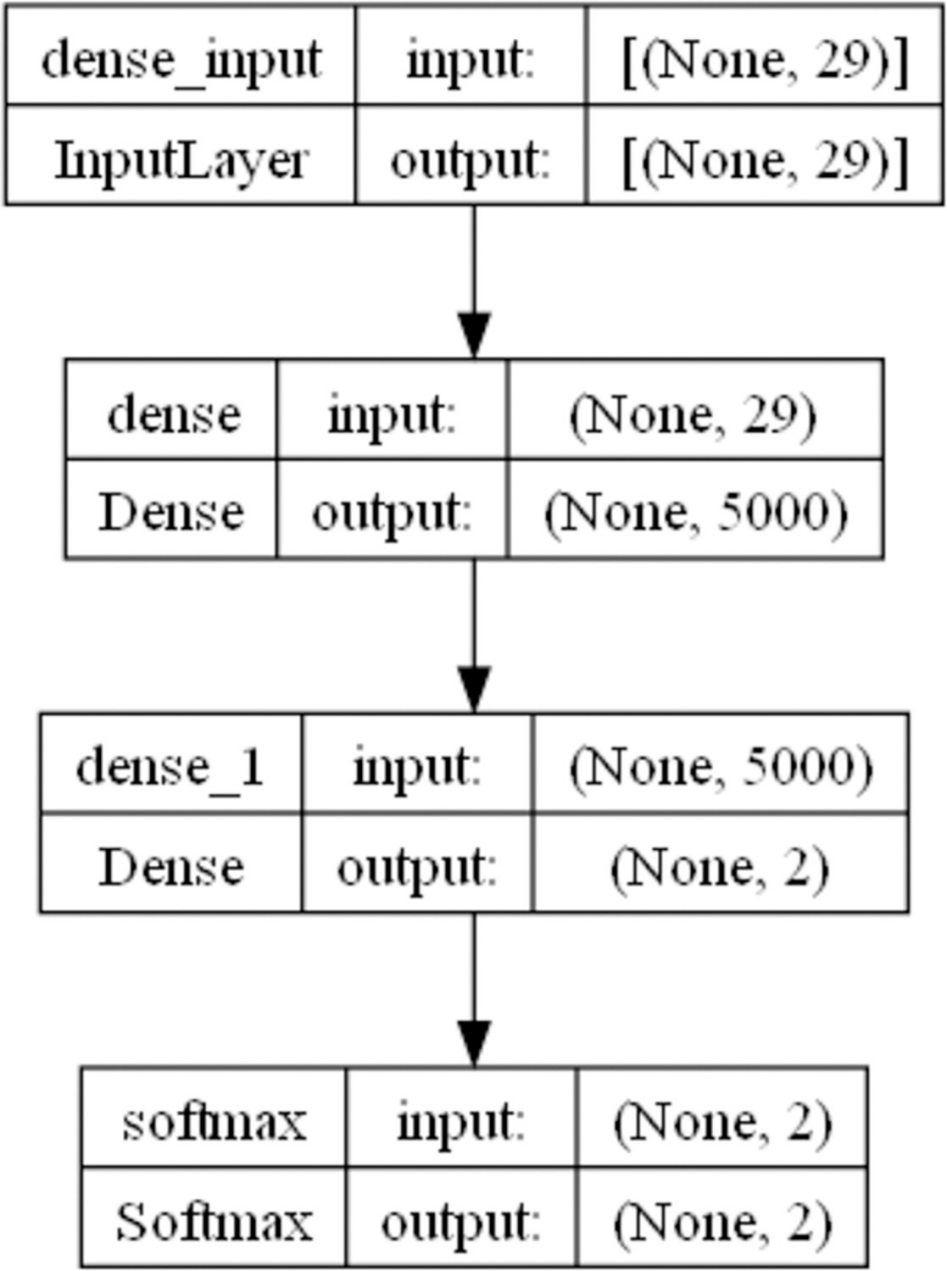
Structure diagram of width neural network.

The neural network model can effectively handle nonlinear classification problems, and deepening the neural network can improve the model’s generalization ability. In order to achieve better fitting results, the second solution in this article is to improve the width of the neural network to improve the prediction accuracy of the model. The model consists of 2 hidden layers and 2 relu layers, which are then output as category variables through the softmax layer. The calculation formula for a wide neural network is as follows:

$$x_{k}^{\left(1\right)}=f_{k}^{\left(1\right)}(v_{i},a_{i})=relu(\sum_{j=1}^{n_{1}}\omega_{k,j}^{\left(1\right)}v_{i,j}+\omega_{k,n_{1}+1}^{\left(1\right)}a_{i}+\omega_{k,n_{1}+2}^{\left(1\right)})$$

$$x_{k}^{\left(2\right)}=\sum_{j=1}^{n_{2}}\omega_{k,j}^{\left(2\right)}x_{i,j}^{\left(1\right)}+\omega_{k,n_{2}+1}^{\left(2\right)}$$

$$P(y_{i}=l)=\frac{\exp(x_{l}^{\left(2\right)})}{\sum_{k=1}^{L}\exp(x_{k}^{\left(2\right)})}$$

$$l=\arg\;\underset{j}{\max}\;p(y_{i}=j)$$

$$n_{1}\geq 1000$$ (7)

where *n*_1_ is the number of neurons in the first layer. In the width neural network, this paper specify that its value is at least greater than 1000 dimensions, *n*_2_ is the number of neurons in the second layer. This is a binary classification problem, therefore *n*_*2*_ = 2. *ω*^(1)^ is the coefficient of the first layer model, *ω*^(2)^ is the coefficient of the second layer model. *v* is the input variable, *a* is the amount of each transaction, *v*_*i*_ is the data in the training set, *x*^(1)^ is the output of the first hidden layer, *x*^(2)^ is the output of the second hidden layer, *f* is the nonlinear transformation function of the hidden layer, *L* is the number of categories (Take *L* as 2 in this article), *b* is the bias term. The *relu* is a nonlinear activation function. Fig 3 is the structural diagram of the width neural network in this article.

### Fusion neural network

**Fig 4:**
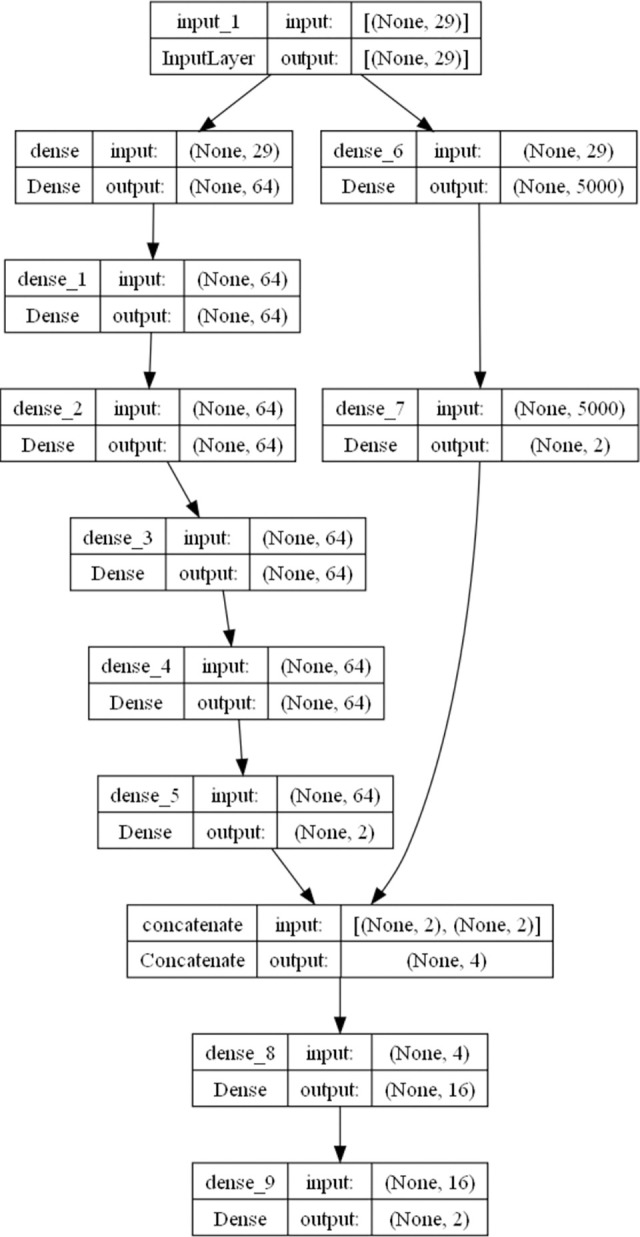
Structure diagram of a fusion neural network.

In order to further improve the recognition accuracy of the model, this paper combine deep neural networks with width neural networks. First, after normalization, the input is processed through a deep neural network and a width neural network, respectively. Second, the outputs of the deep and width neural networks are merged into a vector, which is processed through a hidden layer and a softmax layer to output as the class variables of the neural network. this paper refer to it as a fusion neural network, and the structure of the fusion neural network is as Fig 4.

The calculation formula of the deep neural network in the fusion neural network is as follows:

$$x_{k}^{\left(d,1\right)}=f_{k}^{\left(d,1\right)}(v_{i},a_{i})=relu(\sum_{j=1}^{n_{1}}\omega_{k,j}^{\left(d,1\right)}v_{i,j}+\omega_{k,n_{1}+1}^{\left(d,1\right)}a_{i}+\omega_{k,n_{1}+2}^{\left(d,1\right)})$$

$$x_{k}^{\left(d,2\right)}=f_{k}^{\left(d,2\right)}(x_{i}^{\left(d,1\right)})=relu(\sum_{j=1}^{n_{2}}\omega_{k,j}^{\left(2\right)}x_{i,j}^{\left(d,1\right)}+\omega_{k,n_{2}+1}^{\left(d,2\right)})$$

……

$$x_{k}^{\left(d,6\right)}=f_{k}^{\left(d,6\right)}(x_{i}^{\left(d,5\right)})=relu(\sum_{j=1}^{n_{6}}\omega_{k,j}^{\left(d,6\right)}x_{i,j}^{\left(d,5\right)}+\omega_{k,n_{6}+1}^{\left(d,6\right)})$$ (8)

The calculation formula of width neural network in fusion neural network is as follows:

$$x_{k}^{\left(w,1\right)}=f_{k}^{\left(w,1\right)}(v_{i},a_{i})=relu(\sum_{j=1}^{n_{7}}\omega_{k,j}^{\left(w,1\right)}v_{i,j}+\omega_{k,n_{7}+1}^{\left(w,1\right)}a_{i}+\omega_{k,n_{7}+2}^{\left(w,1\right)})$$

$$x_{k}^{\left(w,2\right)}=f_{k}^{\left(w,2\right)}(x_{i}^{\left(w,1\right)})=relu(\sum_{j=1}^{n_{8}}\omega_{k,j}^{\left(w,2\right)}x_{i,j}^{\left(w,1\right)}+\omega_{k,n_{8}+1}^{\left(w,2\right)})$$

$$n_{7}\geq 1000$$ (9)

The calculation formula of neural network fusion is as follows:

$$x^{\left(f,1\right)}=x^{\left(d,6\right)}⊕x^{\left(w,2\right)}$$

$$x_{k}^{\left(f,2\right)}=f_{k}^{\left(f,2\right)}(x_{i}^{\left(f,1\right)})=relu(\sum_{j=1}^{n_{9}}\omega_{k,j}^{\left(f,2\right)}x_{i,j}^{\left(f,1\right)}+\omega_{k,n_{9}+1}^{\left(f,2\right)})$$

$$x_{k}^{\left(f,3\right)}=f_{k}^{\left(f,3\right)}(x_{i}^{\left(f,2\right)})=softmax(\sum_{j=1}^{n_{10}}\omega_{k,j}^{\left(f,3\right)}x_{i,j}^{\left(f,2\right)}+\omega_{k,n_{10}+1}^{\left(f,3\right)})$$ (10)

where ⊕ stands for splicing two vectors, *d* stands for deep neural network, *w* stands for width neural network and *f* stands for fusion neural network. Softmax function is the category output function of neural network, and its definition is as follows:

$$softmax(x)=[\frac{exp(x^{1})}{\sum_{k=1}^{L}exp(x^{k})},\frac{exp(x^{2})}{\sum_{k=1}^{L}exp(x^{k})},\ldots \ldots ,\frac{exp(x^{L})}{\sum_{k=1}^{L}exp(x^{k})}]$$ (11)

### Model training

The training of the model in this paper is equivalent to minimize the cross entropy loss, as follows:

$$\underset{\omega }{\min}\;Loss=-\sum_{i=1}^{M}\sum_{j=1}^{2}y_{i,j}\log(p_{i,j})$$ (12)

where *p*_*i*,*j*_ is the probability of taking the *j*-th category from the *i*-th sample (Calculated forward by the neural network model). *y*_*i*,*j*_ is the sample label, which represents whether the *i*-th sample belongs to class *j*. If the value is 1, it represents belonging to that class, and if it is 0, it represents not belonging to that class.

In neural network learning, the Loss function is used to guide model learning. Generally, the smaller its value is, the better the performance of the model is. This means that the fewer errors the model produces, the higher the accuracy of classification. Therefore, when training neural networks, goal of this paper is often to minimize the loss function as far as possible. In order to achieve this goal, this paper need to use some optimization algorithms to adjust the parameters of the model. Here, this paper use the rmsprop algorithm to solve the optimization problem. Rmsprop algorithm [40] is a common variant of gradient descent algorithm. its basic idea is to adjust the learning rate according to the square root of gradient to better adapt to different data inputs. The rmsprop algorithm is very common in deep learning, and this paper will not elaborate on its specific details here. In summary, by adopting this optimization algorithm, this paper can better optimize the performance of the model and obtain more accurate and refined prediction results.

### Model evaluation criteria

For the binary classification problem with balanced data sets, accuracy is a useful evaluation metric.At the same time, for any binary classification problem, the F1 score is also a useful evaluation metric. Therefore, two evaluation indicators are used to evaluate the performance of the prediction model in this article, namely accuracy and F1 score [41].

The calculation formula for the accuracy of the classification model is as follows:

Accuracy=(TP+TN)/(TP+TN+FP+FN) (13)

The formula for calculating the F1 score of the classification model is:

F1=2×Precision×Recall/(Precision+Recall) (14)

The precision score calculation formula for the classification model is:

Precision=TP/(TP+FP) (15)

The formula for calculating the recall score of the classification model is:

Recall=TP/(TP+FN) (16)

where *TP* is the number of true cases, *TN* is the number of true negative cases, *FP* is the number of false positive cases, and *FN* is the number of false negative cases.

## Numerical experiment

### Data set

**Fig 5:**
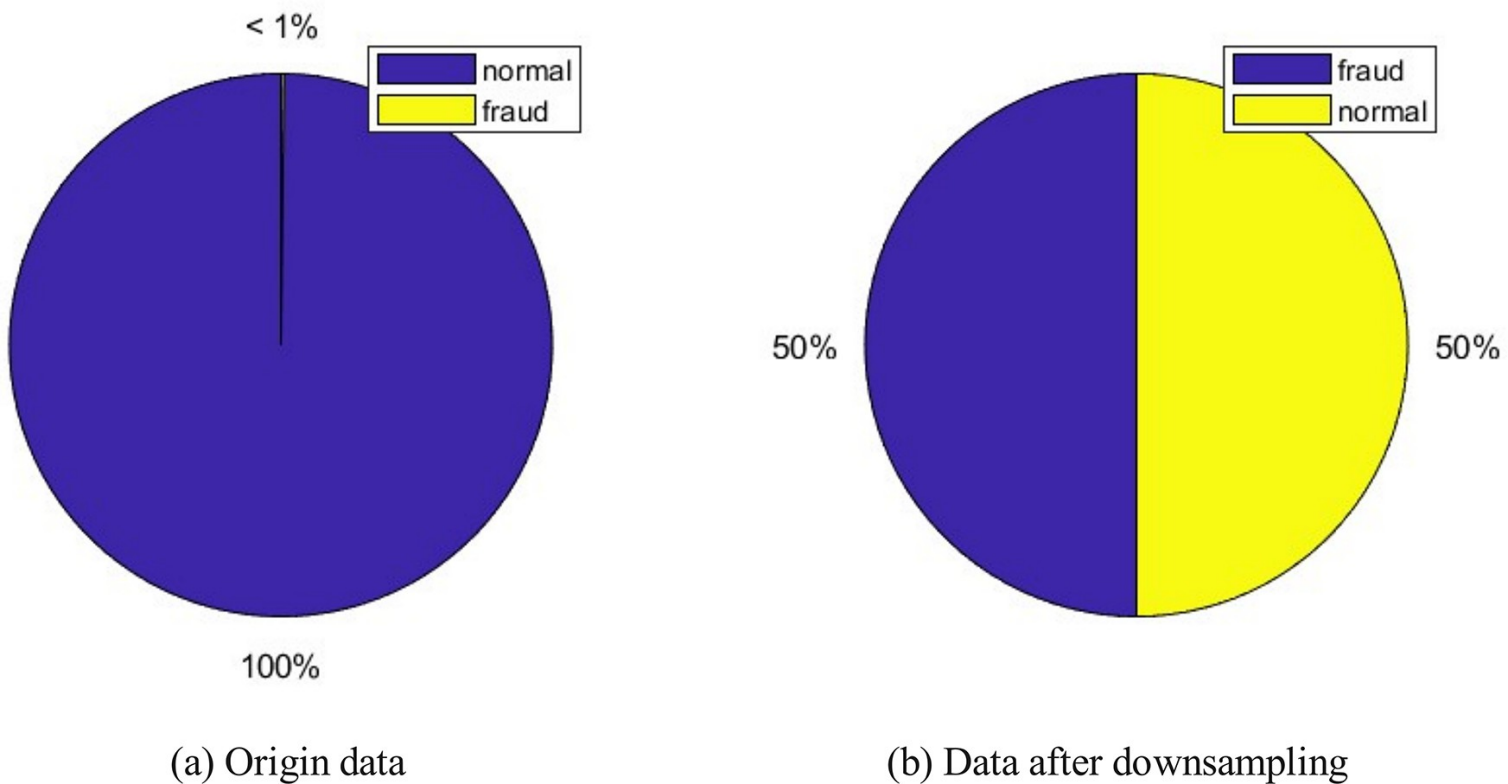
(a) Proportion of normal transactions and fraudulent transactions of raw data. (b) The proportion of normal transactions and fraudulent transactions of the data after down sampling.

The data set [42] used in this article contains transaction information from European cardholders using credit cards in September 2013. This data set shows transactions that occurred within two days, with 492 fraud cases out of 284807 transactions. The data set is highly imbalanced, with fraud accounting for 0.172% of all transactions (see Fig 5). The original dataset has undergone desensitization and PCA processing. 28 anonymous variables are the principal components obtained by PCA, and the only variables that have not been processed by PCA are *time* and *amount*. *Time* is the interval between each transaction and the first transaction in the data set, measured in seconds; *amount* is the transaction amount, *class* is a Categorical variable, which is 1 in case of fraud, and 0 otherwise. We first randomly downsample the non-fraud data in the dataset, as the dataset is imbalanced and the non-fraud data greatly outweighs the fraud data. After downsampling, the categories of the dataset in this article are in a balanced state. Subsequently, 80% of the data was randomly sampled as the training set, and 20% as the testing set. Table 1 is an example of this data. When inputting data into a neural network for calculation, each dimension of the data is subjected to corresponding normalization processing.

**Table 1:** Example of the data set used in this article.

Time	v_1_	v_2_	v_3_	…	v_28_	amount	class
0	‒1.35	‒0.07	2.53	…	‒0.02	149.62	0
1	1.19	0.26	0.16	…	0.01	2.69	0
2	‒1.35	‒1.34	1.77	…	‒0.05	378.66	0

**Fig 6:**
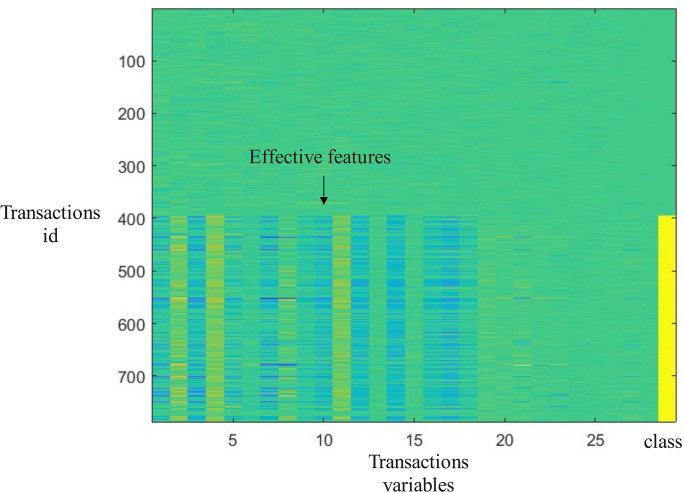
Feature distribution on the training set of this data set.

Fig 6 shows the feature distribution on the training set of this data set, and different colors represent different values. On the far right of the picture are category variables, yellow represents fraudulent transactions, and green represents normal transactions. The horizontal axis is the attributes and fraud categories of each transaction. This paper plot each feature of each transaction record according to its value. The closer the color is to green, the smaller the value, and the closer the color is to yellow, the larger the value. The vertical axis of the graph is the transaction id, and the upper part of the graph is normal transactions, while the lower part is fraudulent transactions. From the graph, it can be seen that there are some differences in the left features between the data in the upper part and the data in the lower part. Therefore, these features can be used as effective features for fraud detection.

**Fig 7:**
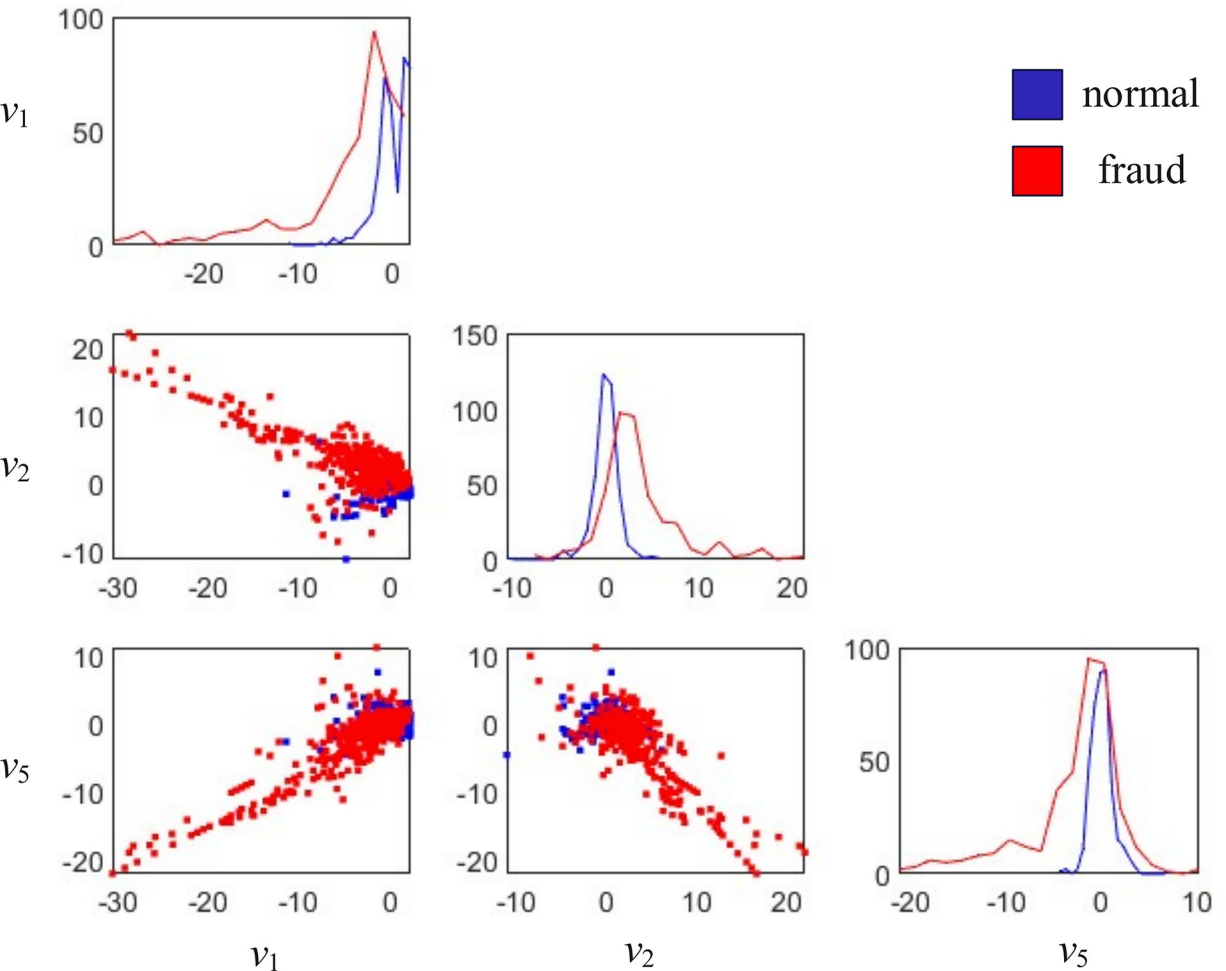
Scatter plot of the first, second, and fifth attributes of credit card transaction records.

This paper extract the first, second, and fifth attributes and group the data based on the label category. Draw their scatter plots and histograms to partially showcase the characteristics of this data, as shown in Fig 7. As can be seen from the figure, there are different distributions of the three variables *v*_1_, *v*_2_, and *v*_5_ for normal data and fraudulent data. These three variables can be used to predict whether there is fraudulent behavior on credit cards.

### Analysis of influencing factors

**Fig 8:**
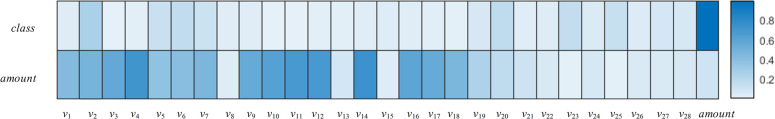
Results of correlation analysis.

This article first studies the correlation between each credit card attribute and whether it is fraudulent. The calculation formula for the correlation coefficient is as follows:

$$r(v,y)=\frac{\left|Cov(v,y)\right|}{\sqrt{Var(v)Var(y)}}$$ (17)

where *Cov* represents the covariance of two variables, and *Var* represents the variance of the variable [43]. This paper visually display the calculation results in Fig 8, and it can be seen that some attributes (*v*_1_, *v*_2_, *v*_5_, *v*_6_, *v*_7_, *v*_8_, *v*_10_, *v*_20_, *v*_23_, *v*_25_) have a certain correlation with whether fraud occurs. These attributes highly correlated with the label can be used to detect whether there is fraud on the credit card transaction.

**Fig 9:**
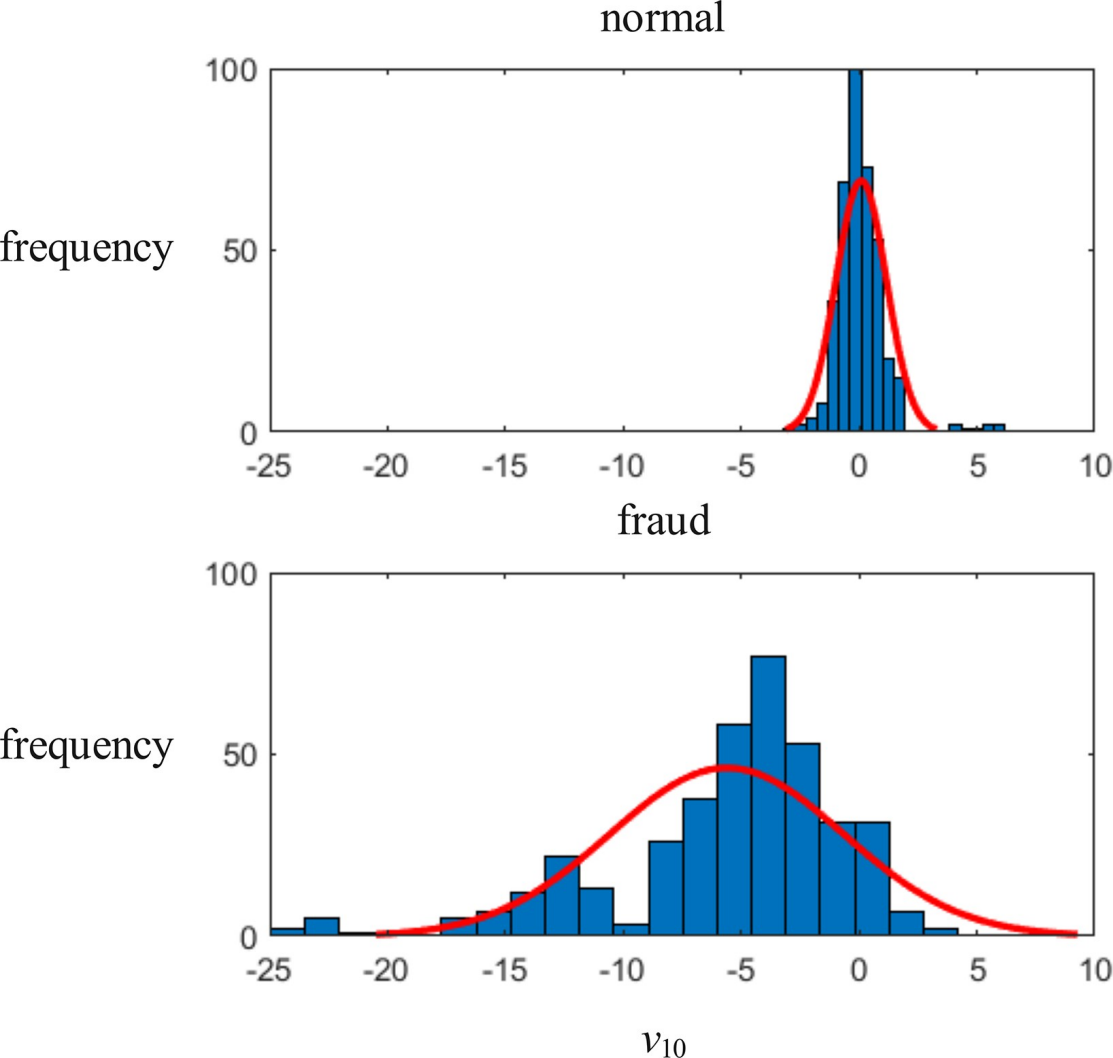
Distribution of the 10-th attribute of fraudulent data and normal data. The upper part is the distribution of the tenth variable of normal data, and the lower part is the distribution of the tenth variable of fraudulent data.

This paper take the 10-th attribute of a credit card as an example to analyze the relationship between various variables and output variables in the data set. We draw a distribution of data labeled as fraudulent and a distribution of data labeled as normal, as shown in Fig 9. As can be seen from the figure, fraudulent transactions and normal transactions have different distributions on the 10-th variable. Therefore, this feature can be considered as an effective variable for fraud identification.

To further understand the impact of each input on the final result, this article uses interpretable AI technology to analyze the data. This article uses Logstic regression to fit the model [44]. The parameters of each variable in the model can reflect the importance of each variable to a certain extent. The greater the weight, the higher the correlation between the variable and the label. This article shows the weights of each input, as shown in the Table 2.

**Table 2:** Parameters of the logistic model.

Parameter	w1	w2	w3	w4	w5	w6	w7
Value	0.04	-0.25	0	0.79	-0.03	-0.01	0.35
Parameter	w8	w9	w10	w11	w12	w13	w14
Value	-0.77	-0.34	-0.69	0.53	-0.96	-0.37	-1.47
Parameter	w15	w16	w17	w18	w19	w20	w21
Value	-0.1	-0.72	-0.38	-0.12	-0.16	0.23	-0.08
Parameter	w22	w23	w24	w25	w26	w27	w28
Value	0.41	-0.66	-0.34	-0.09	-0.7	0.11	0.4
Parameter	w29						
Value	0						

As can be seen from the table, there is a high correlation between some of the labels and the final label, such as w8, w10, w11, w12, w14, w16, w23.

### Experimental results

This paragraph mainly describes the hyperparameters and experimental results of deep neural networks. When inputting data into a neural network, normalization is the first step. Set the dimensions of the all hiden layers to 64, and the dimensions of the sixth layer to 2. Finally, through the softmax layer, the output is a category variable. The nonlinear activation function uses the relu function. The maximum number of iterations is set to 100, and the batch size is set to 64. In this paper, numerical experiments are carried out, and the average accuracy is 94.92% and the average F1 score is 94.44%, the recall score is 90.43%, the precision score is 98.83%.

This paragraph mainly describes the hyperparameters and experimental results of the width neural network.The first layer dimension of the width neural network is set to 5000 dimensions, and the second layer dimension is set to 2. After passing through the softmax layer, the output is a category variable. When inputting data into a neural network, normalization is the first step. The maximum number of iterations is set to 1000, and the batch size is set to 64. Using a wide neural network and conducting 10 calculations repeatedly. The average accuracy of experiments is 95.43%, and the average F1 score is 95.03%, the recall score is 91.49%, the precision score is 98.85%.

This paragraph mainly describes the hyperparameters and experimental results of the fusion network. Before entering the deep neural network and the wide neural network for computation, the data needs to be normalized first. Set the number of layers for the deep neural network to 6, with an output length of 64 for all hidden layers. The last level output dimension of the deep branch is 2. The first layer dimension of the width neural network is 5000, and the second layer length is 2. Splice the output of the width neural network and the depth neural network together, pass through a hidden layer with a length of 16, and then pass through a dense and softmax layer to output as a category variable. This paper set the batch size as 64.

Some of the hyperparameters in this article, such as the number of hidden units per layer in deep networks and the number of hidden units in wide networks, are mainly optimized based on 5-fold cross-validation on the training set. Regarding the choice of activation function, we have chosen the relu function for all hidden layers because it can effectively prevent the problems of vanishing or exploding gradients. The number of layers and batch size of the neural network are mainly selected with reference to relevant paper and experience on neural networks [45].

**Fig 10:**
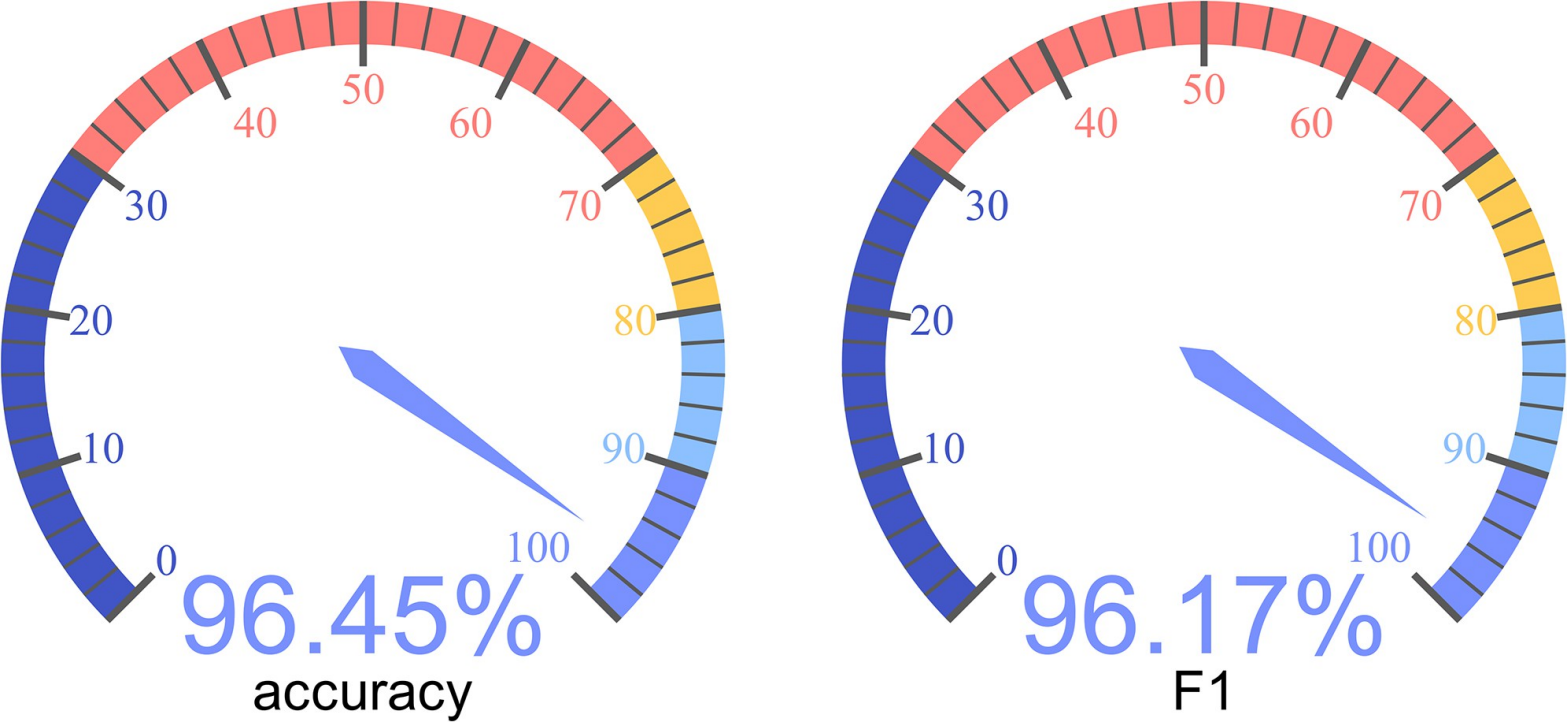
Test results of fused neural network.

On the test set, the experimental results of this method are shown in Fig 10.

Table 3 shows the statistical analysis of 10 experiments on the fusion neural network. The average accuracy and F1 score were 96.45% and 96.17%, respectively. The standard deviations of the two indicators in each experiment were 0.39% and 0.34%.

**Table 3:** Recognition results of 10 experiments with fusion neural network.

Experiment id	Accuracy	F1
1	95.96%	96.15%
2	96.43%	96.03%
3	96.95%	96.45%
4	96.45%	96.17%
5	95.94%	95.60%
6	96.95%	96.70%
7	96.45%	96.13%
8	96.95%	96.70%
9	95.94%	95.65%
10	96.44%	96.13%
Average	96.45%	96.17%
Std	0.39%	0.34%

It can be seen that the average accuracy of the fusion neural network is 96.44%, and the average F1 score is 96.17%, the recall score is 93.62%, the precision score is 98.88%.

### Comparison with other models

This article compares the proposed method with machine learning algorithms. Firstly, the accuracy of the KNN algorithm [9] is compared as shown in Table 4. The number of neighbors in the KNN algorithm is set to 5, and the distance measurement is based on Euclidean distance. The weight is weighted using the inverse proportion of distance. Secondly, this paper compared the accuracy of the decision tree [14]. The maximum depth of the decision tree is set to 15, the splitting index is used as the Gini index, and the maximum number of features is set to 50. Finally, this paper compared the accuracy of Bayesian algorithm [15].

**Table 4:** Comparison between this method and machine learning method.

Model	accuracy	F1	Recall	Precision
Fusion neural network	96.44%	96.17%	93.62%	98.88%
Deep neural network	94.92%	94.44%	90.43%	98.83%
Width neural network	95.43%	95.03%	91.49%	98.85%
KNN	92.5%	92.31%	90%	94.73%
Decision tree	90.5%	90.64%	92%	89.32%
Bayesian	89%	87.91%	80%	97.56%

From the above analysis of results, it can be concluded that the method (deep neural network, width neural network, fusion neural network) proposed in this paper is better than machine learning algorithms such as KNN and decision trees, Bayes, etc. In addition, it is worth noting that compared with the width neural network and the depth neural network, the fusion neural network proposed in this paper has made some progress to a certain extent.

To further verify the performance of the fusion network, this paper compares the model with deep learning models and ensemble models. The ensemble models this paper want to compare include: random forest and adaboost [25]. The deep learning models this paper want to compare include: long short-term memory network (LSTM) and convolutional neural network (CNN) [46]. The number of weak classifiers in the random forest is set to 100, the segmentation evaluation metric is set to gini, the maximum depth is set to 3, and the maximum number of features is set to 5. The base learner of adaboost is set to decision tree, the number of base learners is set to 100, the learning rate is set to 1, and the training algorithm is SAMME. The number of hidden layer units in the LSTM model is set to 32. This paper treat each feature as a time step and input it into a recurrent neural network. The hidden layer activation function uses relu, and the last layer activation function uses softmax. The CNN model has 4 one-dimensional convolutional layers, each with 8 channels. The activation function is relu, and the last layer is a fully connected layer with softmax activation function. The comparison results are shown in Table 5. From Table 5, it can be seen that the detection F1 score of this model is slightly higher than that of deep learning models and ensemble learning models.

**Table 5:** Comparison with ensemble models and deep learning models.

Model	accuracy	F1	Recall	Precision
Fusion neural network	96.44%	96.17%	93.62%	98.88%
Random foresr	92.5%	92.06%	87%	97.75%
Adaboost	95%	95%	95.01%	95%
LSTM	95.93%	95.65%	93.61	97.77%
CNN	95.43%	95.08%	92.55%	97.75%

### Time and space complexity analysis

The time complexity of forward calculation of the model is an important indicator, because the time of forward calculation has a great impact on the successful deployment of the model. For a fair comparison, both models were implemented on Huawei computers, with a CPU frequency of 1.9GHz and 16GB of memory. Table 6 shows the Time complexity comparison between the model in this paper and several models to be compared.

**Table 6:** Time complexity comparison between method of this paper and machine learning method. (Calculation time for 100 forward inferences).

Model	Time (s)
Deep neural network	0.041
Width neural network	0.039
Fusion neural network	0.039
KNN	0.04
Decision tree	0.001
Bayesian algorithm	0.95
Random forest	0.0045
Adaboost	0.0033
LSTM	0.042
CNN	0.038

The wide neural network model in this article consumes 0.039 seconds for forward inference of 100 data points, while the deep neural network model consumes 0.041 seconds. The calculation time for 100 data points in the fusion neural network inference is 0.039 seconds. Generally speaking, a neural network model with 100 forward inferences less than 1 second can meet the actual deployment requirements [47]. Therefore, the time complexity of the model in this paper can meet the needs of fast calculation.

This paper measures the space complexity of the model by the size of the model file exported from the software. Table 7 shows the test results of space complexity of this model and space complexity of other models. The deep neural network model consumes 273 KB of memory, and the wide neural network model consumes 1894 KB of memory, the memory consumed by the fusion neural neural network is 2170KB. Space complexity of the model in this paper is very small and can be ignored compared with the current computers and servers. Therefore, the space complexity of this model can meet the requirements.

**Table 7:** Space complexity comparison between method of this paper and other method.

Model	Space (KB)
Deep neural network	273
Width neural network	1894
Fusion neural network	2170
KNN	205
Decision tree	9
Bayesian algorithm	2
Random forest	124
Adaboost	134
LSTM	75
CNN	64

## Conclusions

This paper proposes a fusion network model combining deep neural networks and wide neural networks, and applies it to credit card fraud identification. The width neural network proposed in this article achieved an accuracy of 95.43% and an F1 value of 95.03% on the test set. The constructed deep neural network achieved an accuracy of 94.92% and an F1 score of 95.03% on the test set. The fusion neural network constructed in this article achieved an average accuracy of 96.44% and an F1 value of 96.17%. This article compares the method with several traditional machine learning models, including decision trees, KNN, and Bayesian networks. At the same time, this article also compares the model with the ensemble model and deep learning model. From the experimental results, method of this paper has achieved good results. From a time complexity analysis, it takes 0.359 seconds to perform 100 inferences. In terms of spatial complexity, the size of model of this paper can be negligible compared to current computers and servers.

This article combines deep neural networks and wide neural networks to improve detection accuracy. However, there is still a problem that the binding mechanism is not deep enough. In future research, we will delve deeper into the integration mechanism of deep neural networks and wide neural networks.

Currently, the transformer model is a very popular deep learning model. In our future work, we will try to propose a similar credit card fraud identification model to further improve the accuracy of identification. At the same time, we will also conduct experiments on more features and larger-scale data sets.
